# Chairside digital workflow to obtain an optimized 3D model of a partially edentulous mandible

**DOI:** 10.3389/froh.2025.1712600

**Published:** 2026-01-05

**Authors:** Alexandru E. Petre, Sergiu Drafta, Andrei Macris

**Affiliations:** Prosthetic Department, Dental Faculty, Carol Davila University of Medicine and Pharmacy, Bucharest, Romania

**Keywords:** 3D model, digital workflow, intraoral scanner, optical impression, removable partial denture

## Abstract

Intraoral scanners generate accurate optical impressions for dentate areas, but wide edentulous spans remain challenging due to limited stable landmarks and mobile mucosa. This article describes a single-visit, chairside workflow that combines an intraoral scan with a digitized irreversible hydrocolloid impression to obtain an optimized three-dimensional model of a partially edentulous mandible suitable for computer aided design-based removable partial denture fabrication other prosthetically driven procedures. The workflow includes intraoral scanning, conventional alginate impression taking followed by chairside digitization with the same scanner, mesh trimming and inversion to generate a positive mucosal surface, and integration of both datasets through sectioned areas and targeted best-fit alignment. The resulting composite model preserves the dentate part from the impression scan. The composite model accuracy should be validated intraorally a three-dimensional printed try-in. This fully chairside protocol provides a practical approach to producing a working mandibular model for digital prosthodontic applications for patients who require a minimal clinical appointment.

## 1 Introduction

Intraoral scanning (IOS) has become a widely adopted alternative to conventional impression techniques for fixed prosthodontics and short span restorations (1–3). In contrast, full arch scans that traverse broad edentulous spans remain clinically challenging because stable landmarks are sparse and the unattached mucosa is mobile. Early explorations even attempted complete denture workflows with IOS and reported recordings of functional borders (4–7). Yet feasibility has not equated to consistent trueness: accuracy over edentulous arches remains device and protocol dependent, as shown by comparative work on edentulous jaws (8–12).

Beyond landmark scarcity, determinants of full arch IOS accuracy include scanner hardware/software, scanning strategy, the scanned arch, and the use of auxiliary land-marks or scanning aids. Recent studies indicate that device choice and scanning pattern influence trueness/precision (13, 14); artificial landmarks/scanning aids can modulate outcomes (15, 16). Such variability helps explain heterogeneity across reports and motivates hybrid solutions when a mucostatic (pressure free) record of lining mucosa is desired. The problem is particularly pronounced in the partially edentulous mandible, where soft tissue mobility and fewer attached mucosal references challenge stitching and alignment (8, 12, 13).

When a mucostatic record is required—especially with flabby tissue—hybrid methods that integrate an IOS optical impression with a conventional impression have been described (17–19).

A chairside workflow is presented that performs both captures with the same IOS, process meshes and produces an optimized full arch three-dimensional (3D) model in a single visit. Because digitizing the dentate portion of a conventional impression with an IOS may be less reliable at greater depths, the workflow preserves IOS derived dental surfaces and substitutes impression derived mucosal patches—seeking to minimize errors from scanning the dentate region of the impression and deformation of flabby tissues during optical capture (20). The composite model is intended for digital removable partial denture (RPD) framework design and other prosthetically driven tasks (implant planning, surgical guide design) (21–24).

In routine practice, the downstream success of a digitally planned removable partial denture hinges on the fidelity of the source model. Errors created during full arch IOS over edentulous spans can propagate into framework design—altering major connector adaptation, rest seat geometry, and clasp undercut engagement. By combining high-definition dental data from the IOS with stable, impression derived mucosal surfaces, the composite model can improve predictability for path of insertion analysis, relief over mobile mucosa, and definition of finishing lines for major connectors. Such refinements are relevant not only for RPD frameworks but also for prosthetically driven implant placement and surgical guide design, where trueness over extended soft tissue fields is consequential (10–12, 21–24). Operationally, a chairside composite model may decrease laboratory communication loops, reduce adjustment time at insertion, and shorten turnaround—effects that warrant empirical evaluation and could ultimately lower costs for clinics and laboratories (21–24).

We hypothesize—subject to future validation—that composite models generated by this hybrid workflow will:

- Exhibit higher trueness over edentulous mandibular spans than IOS only models;
- Preserve the precision of dentate regions;
- Yield frameworks with improved clinical fit and reduced adjustment time relative to IOS only designs.

The aim of this article is to detail a reproducible, single visit technique that merges an IOS scan with a digitized irreversible hydrocolloid impression to obtain an optimized mandibular 3D model in a partially edentulous patient. This manuscript is intended as a technical workflow description rather than a validation study. A clinical case will be presented as a proof-of-concept to complement this methodological description, with standardized pre- and post-treatment photographic documentation of the definitive prosthesis available.

## 2 Materials and methods

### 2.1 General technique

**Figure 1:**
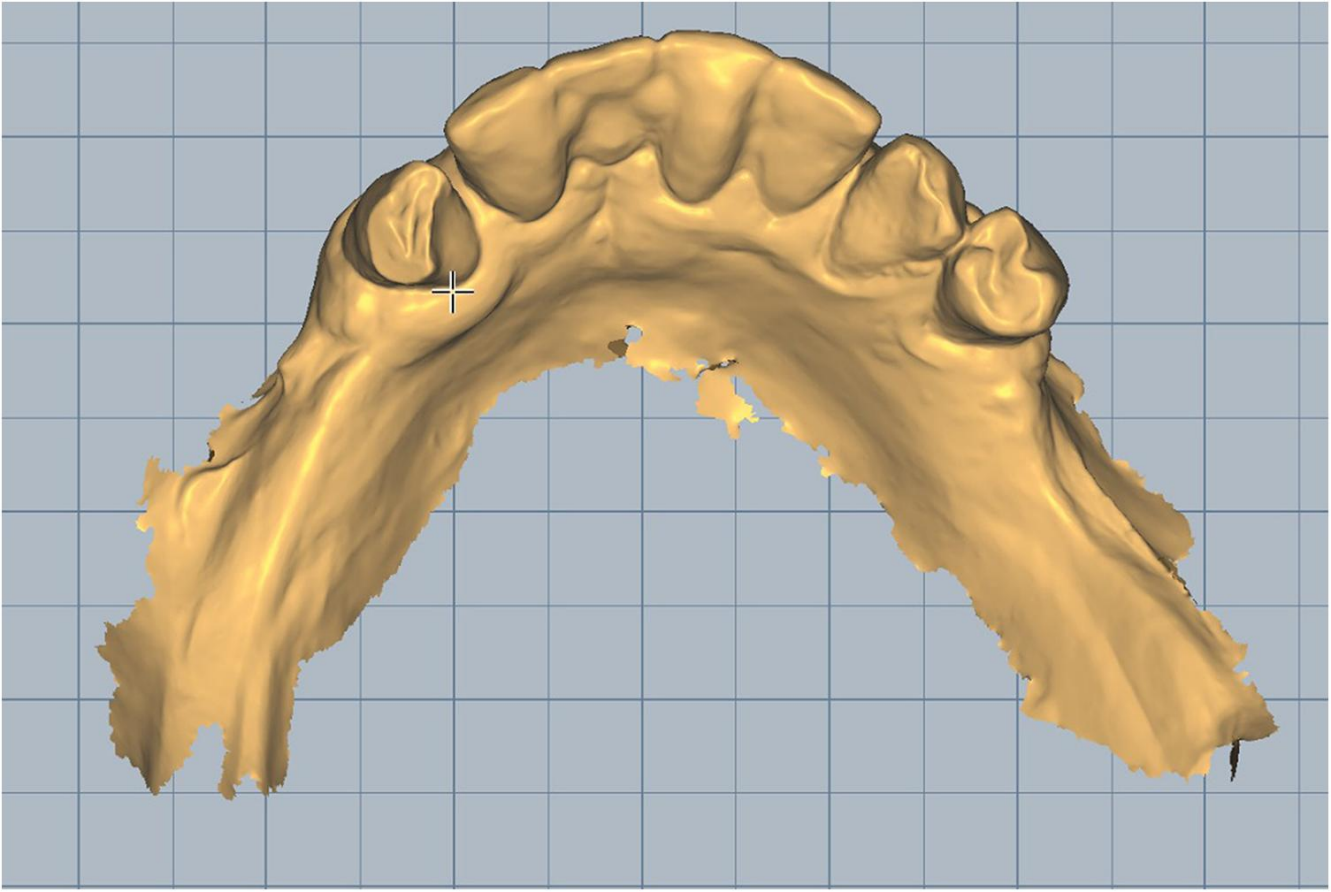
Intraoral scan of lower jaw.

**Figure 2:**
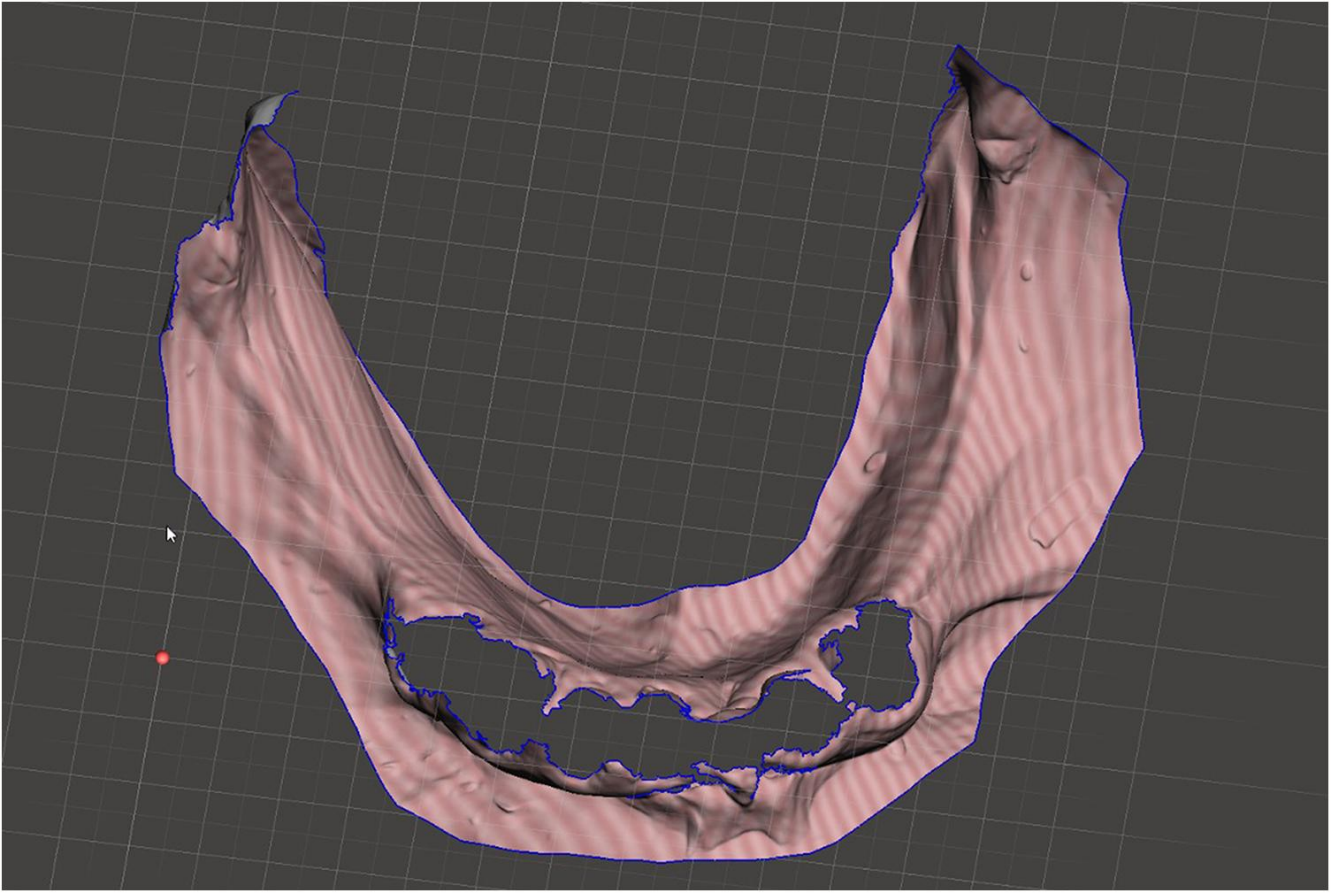
Digitized conventional impression.

**Figure 3:**
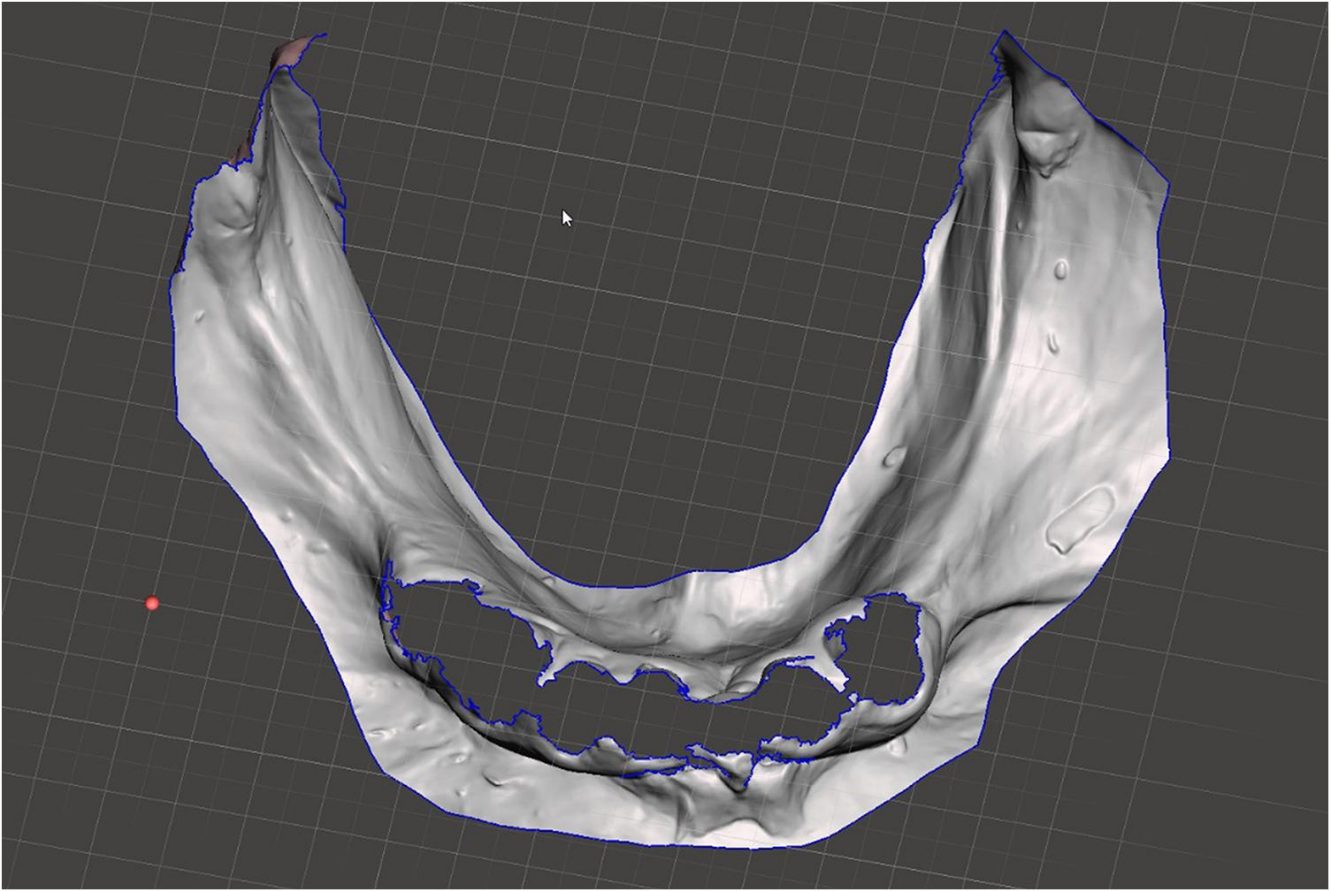
Digitized reversed conventional impression.

**Figure 4:**
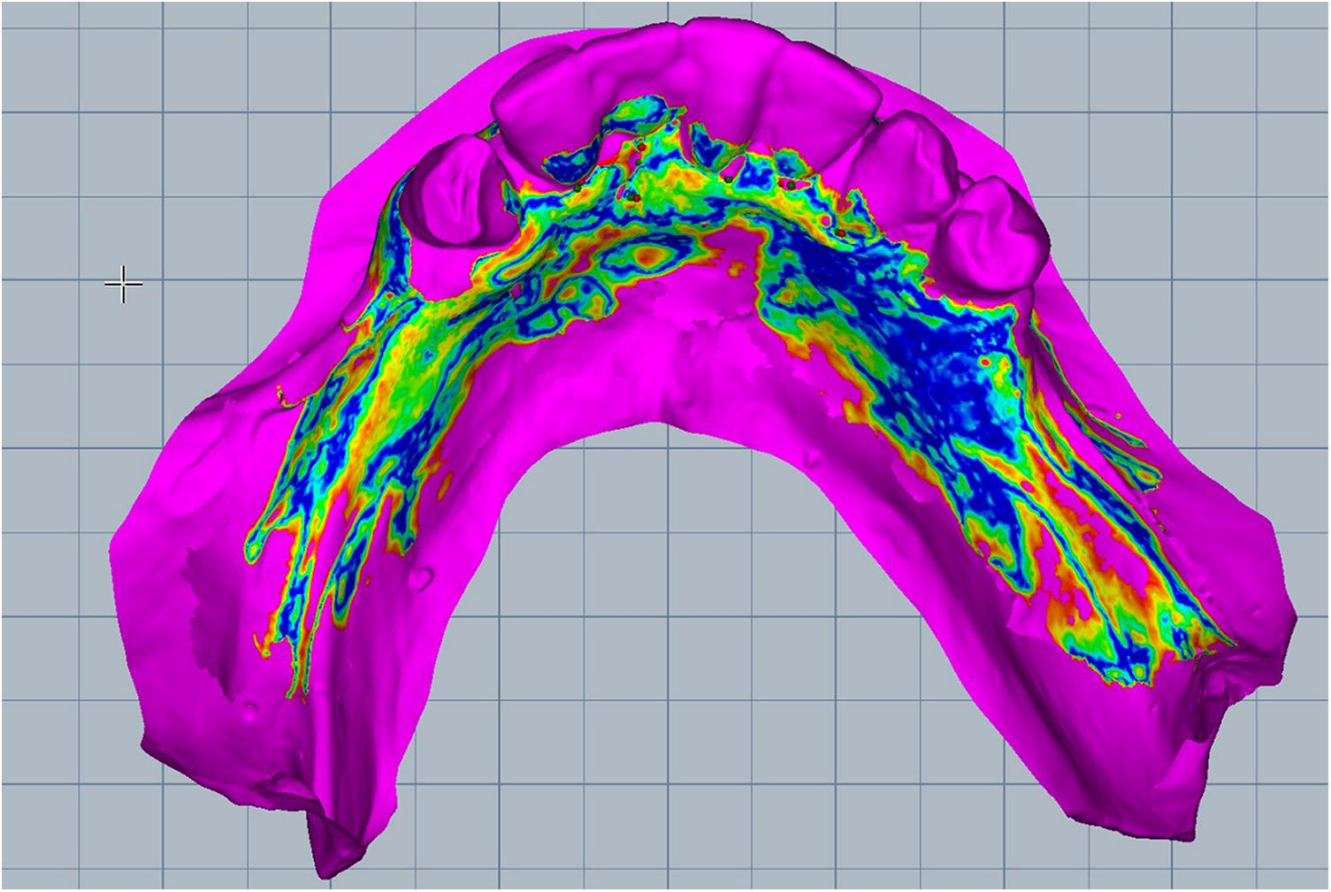
Merged the standard tessellation language files and superimposed both scans.

**Figure 5:**
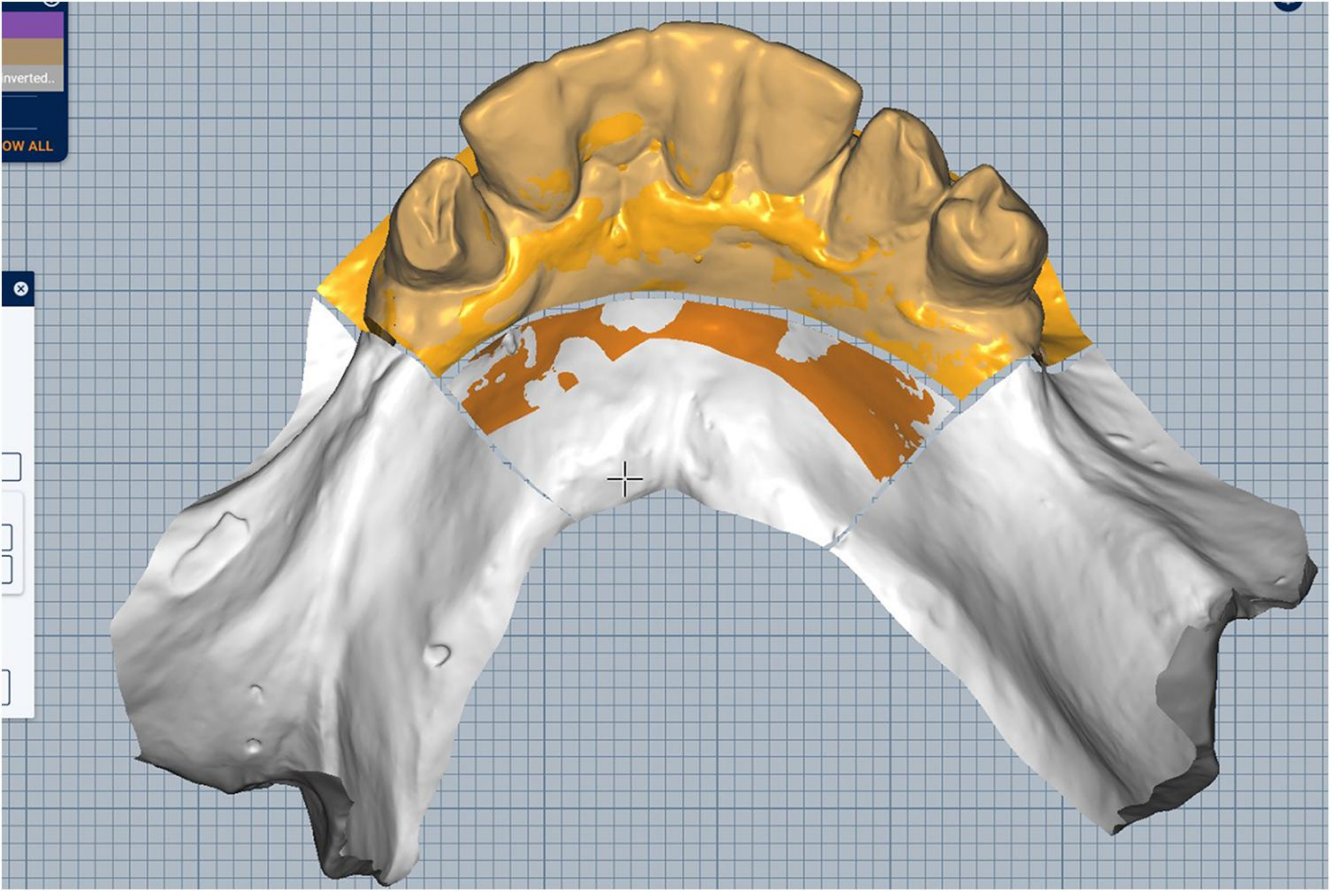
Sectioned areas placed on previously superimposed scans.

1. Scan the mandibular arch with the IOS (3Shape TRIOS 3; 3Shape A/S; Copenhagen K, Denmark) including as much of the edentulous mucosa as possible. Verify continuous scan paths and absence of holes. Export the scan as a standard tessellation language (STL) file and import into a design software program (exocad; exocad GmbH; Darmstadt, Germany) (Figure 1).
2. Make an irreversible hydrocolloid conventional impression (hydrogum 5; Zhermack SpA; Badia Polesine, Italy), in a stock or custom tray. Immediately digitize the impression intraorally scanner-assisted by scanning the impression surface with the same IOS (3Shape TRIOS 3; 3Shape A/S; Copenhagen K, Denmark), no laboratory scanner being required. Export the scan as an STL file and import this file into 3D mesh processing software (Meshmixer3.5.474; Autodesk, Inc, California, San Francisco, USA). (Figure 2).
3. Because the impression is a negative replica, in the 3D mesh processing software, convert it to a positive mucosal surface using mesh operations (e.g., flip face “normal” if needed; (Figure 3).
4. Import both STL files (the IOS scan and the positive mucosal STL file from Step 3) into the design software program (exocad; exocad GmbH; Darmstadt, Germany). Use global registration to roughly align the datasets and perform a best-fit refinement (Figure 4).
5. Create separation areas between dentate core and lateral mucosal patches. Export as a new STL file (Figure 5).

This configuration minimizes computational drift, maintains predictable substitution boundaries, and avoids the unnecessary complexity associated with additional segmentation lines.

**Figure 6:**
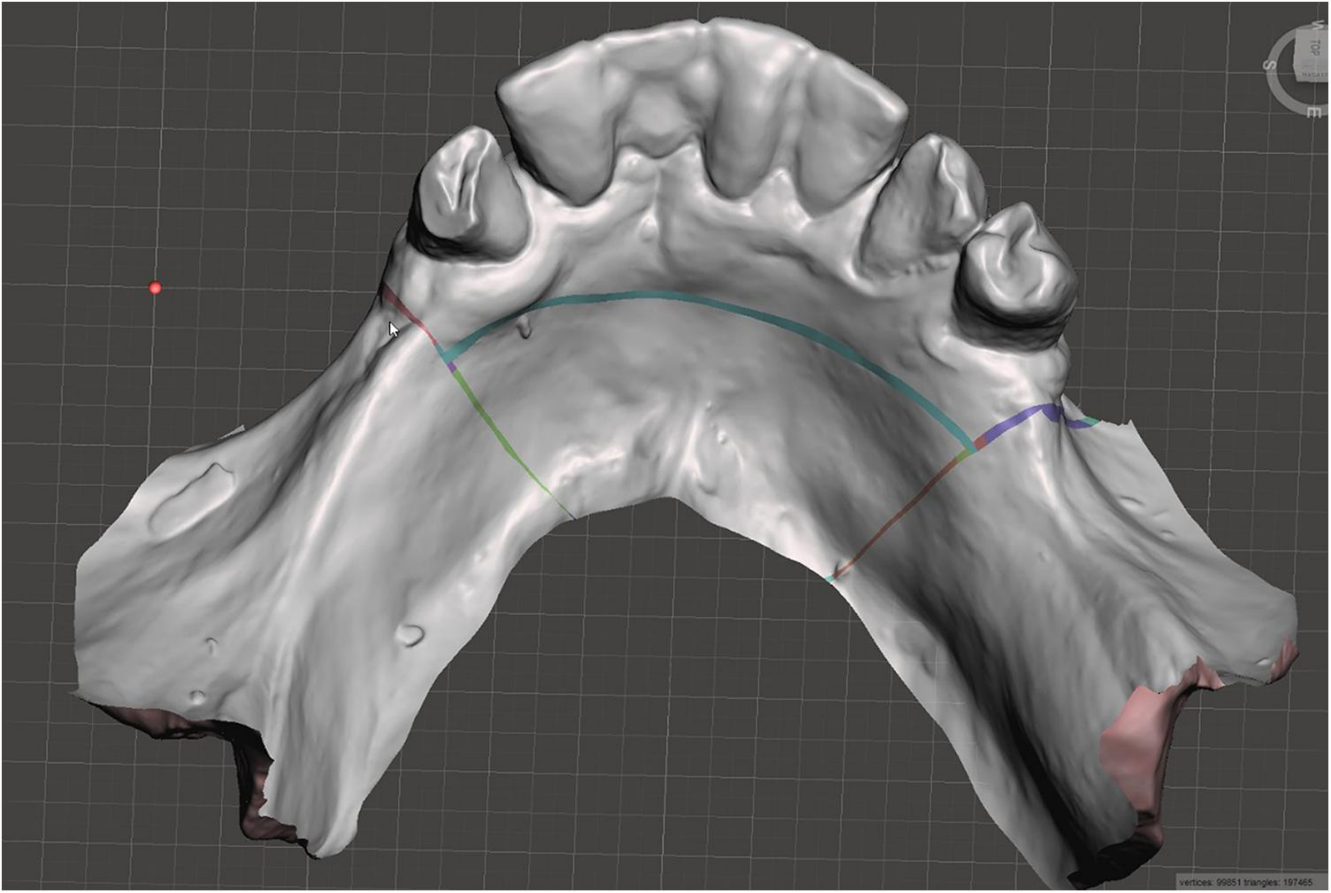
Aligned mucosal gingival area of optical impression with corresponding area of digitized conventional impression.

**Figure 7:**
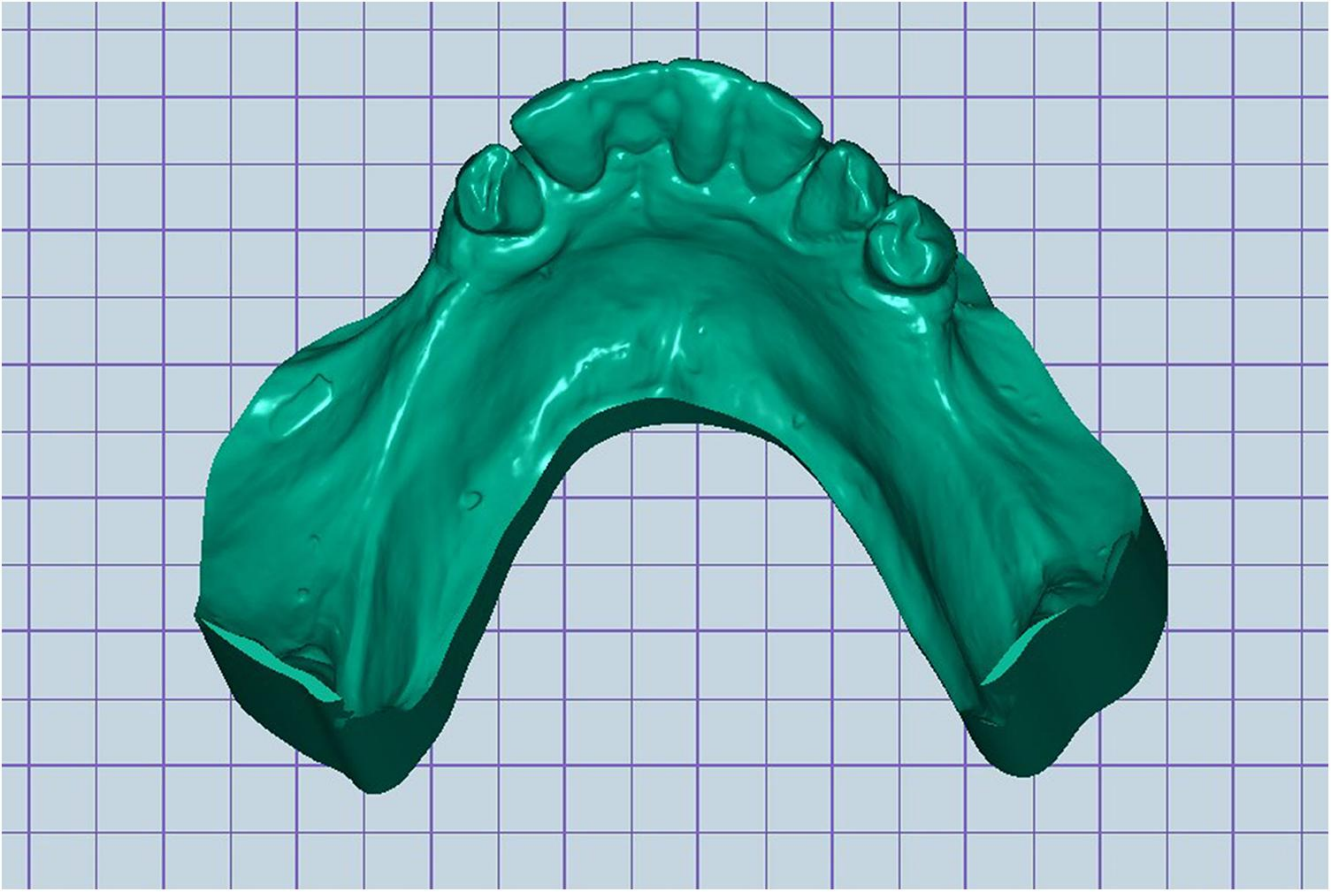
Digital 3D model of lower jaw.

6. In the 3D mesh processing software (Meshmixer3.5.474; Autodesk, Inc, California, San Francisco, USA), locate and re-import the aligned STL file and fuse the border's sectioned IOS mucosal areas with the corresponding left/right segments from the digitized impression using bridge function. This step replaces areas of the IOS mucosa that may be distorted with the impression-derived mucosa. Export as a single STL file (Figure 6).
7. In the design software program (exocad; exocad GmbH; Darmstadt, Germany), locate and import previously STL file and fill the remaining holes between fused sectioned mucosal areas to obtain a single mesh. Inspect for artifacts (holes, self-intersections) and repair if required. Save the final mandibular 3D model as STL file for downstream computer aided design (CAD) software (Figure 7).
8. Quality checks:

Verify the occlusal contact areas and marginal gingiva continuity after fusion.

It is important to emphasize that the irreversible hydrocolloid impression was intentionally recorded in a mucostatic manner. The goal of this impression within the hybrid workflow is not to obtain a functional impression of muco-gingival area but rather to capture a non-displaced static representation of the lining mucosa. Functional muco-gingival remains impression part of the clinical steps performed later during removable partial denture fabrication and is independent of the digital acquisition workflow described here.

Best-fit alignment was deliberately restricted to rigid, dentate landmarks and to pre-segmented soft-tissue patches to avoid incorporating mucosal deformation from the intraoral scan. Global best-fit across the entire mucosa would be inappropriate because intraoral and impression-derived tissues are captured under different conditions. Local alignment confined to stable references ensures that impression-derived mucosal surfaces replace only those IOS regions susceptible to distortion.

### 2.2 Clinical report (proof-of-concept)

**Figure 8:**
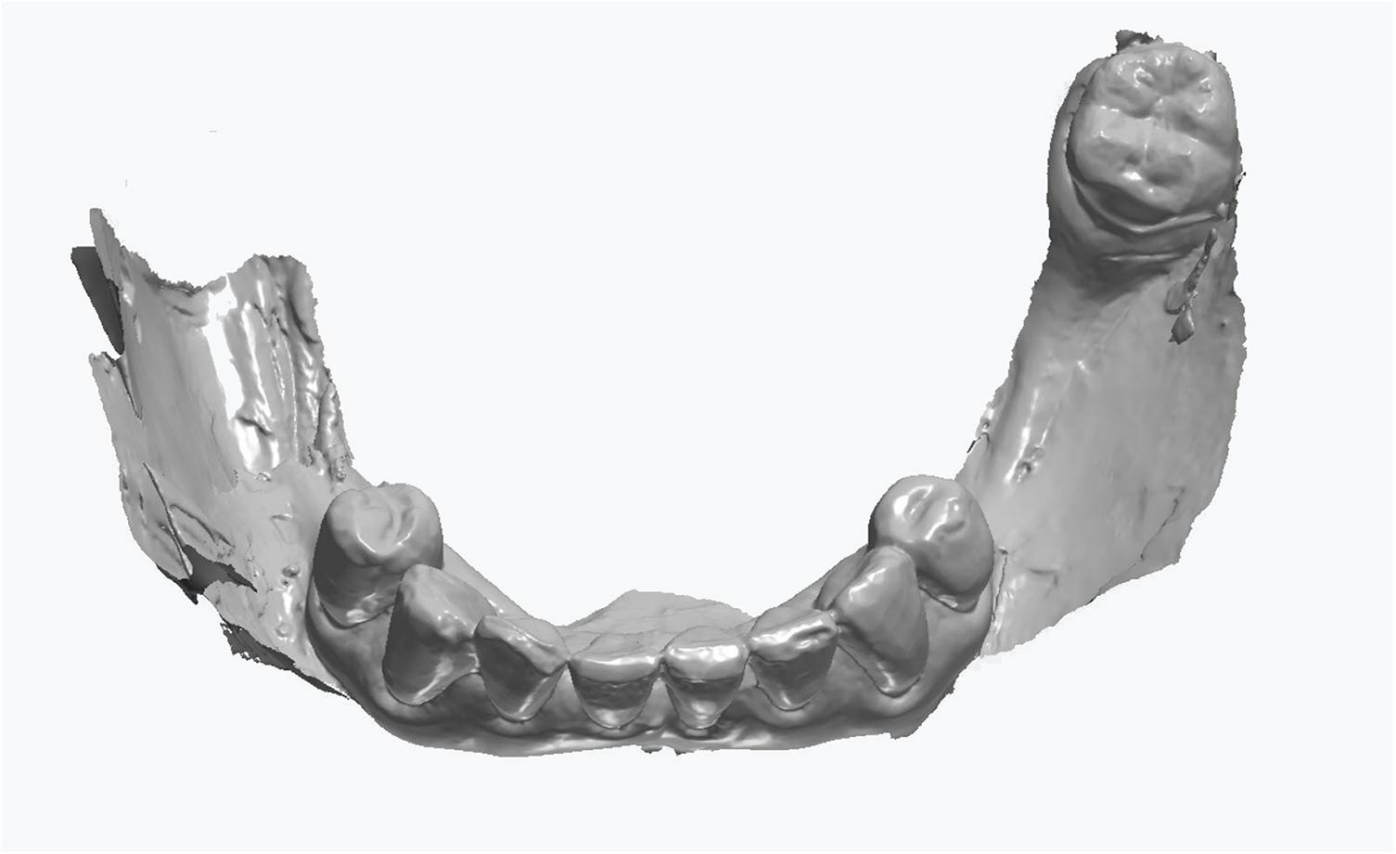
Intraoral scan of lower jaw with attached mucosa of both sides of the mandible and frontal dentate area.

**Figure 9:**
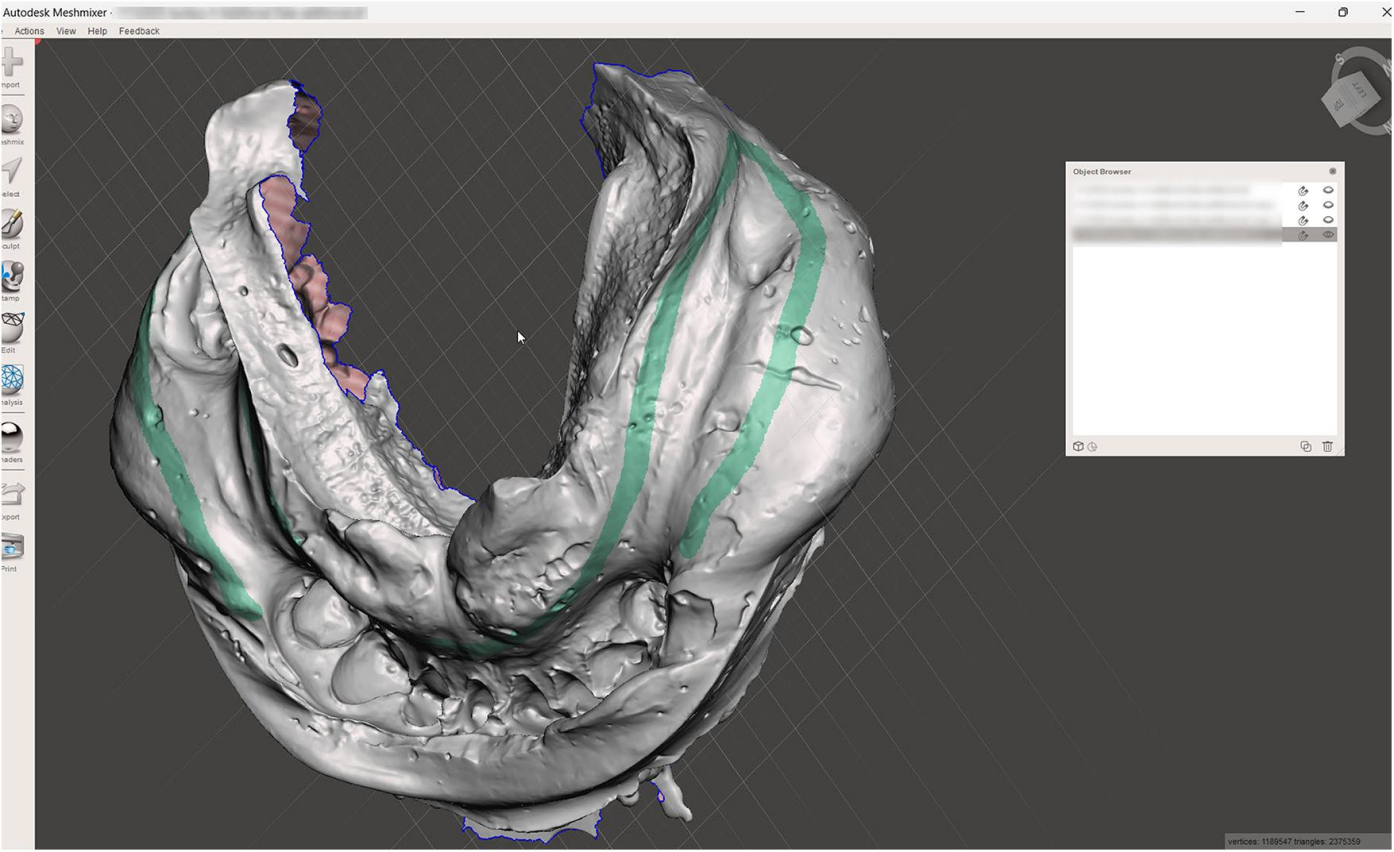
The digitized conventional impression with marked impression borders.

**Figure 10:**
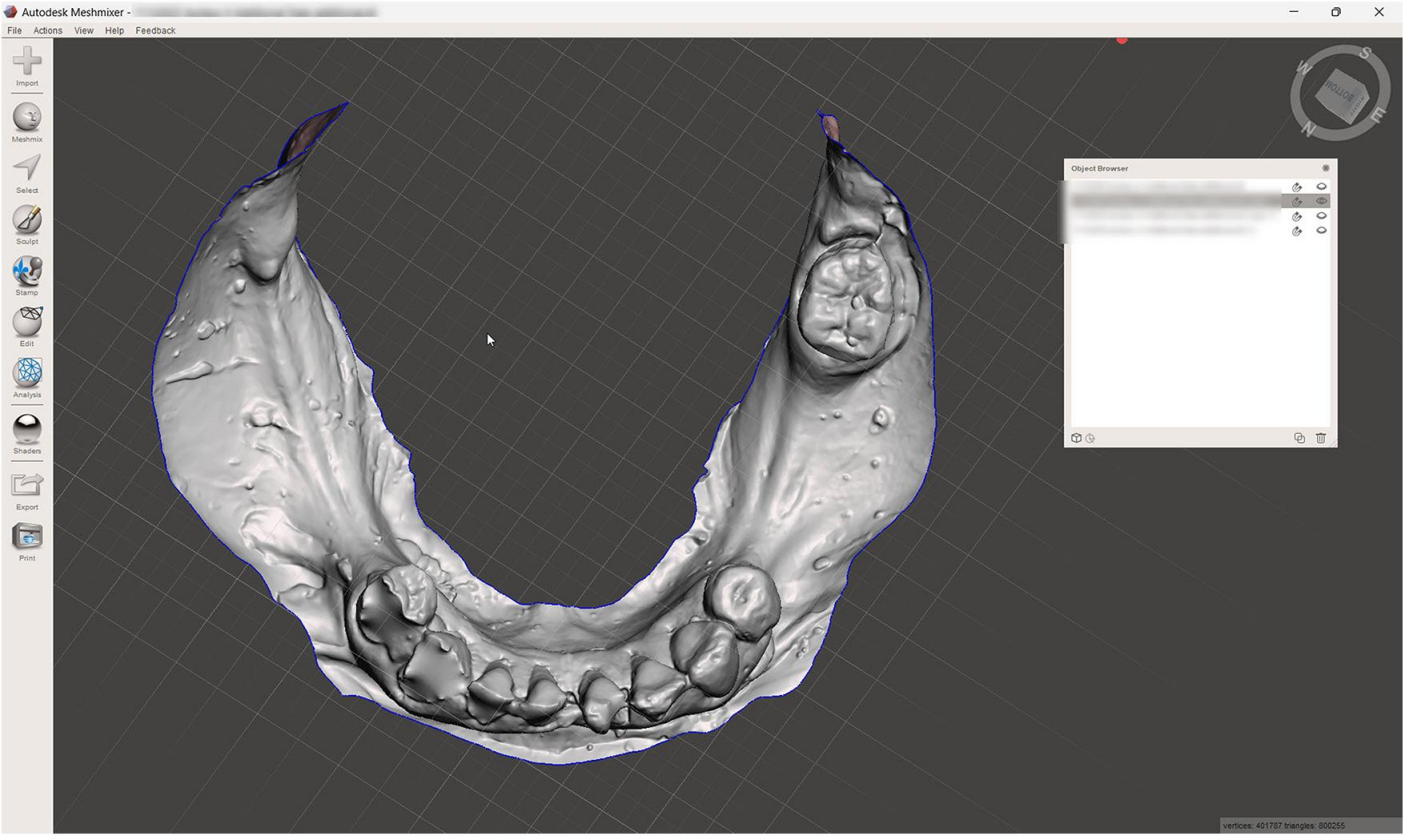
The digitized conventional impression mesh inverted.

**Figure 11:**
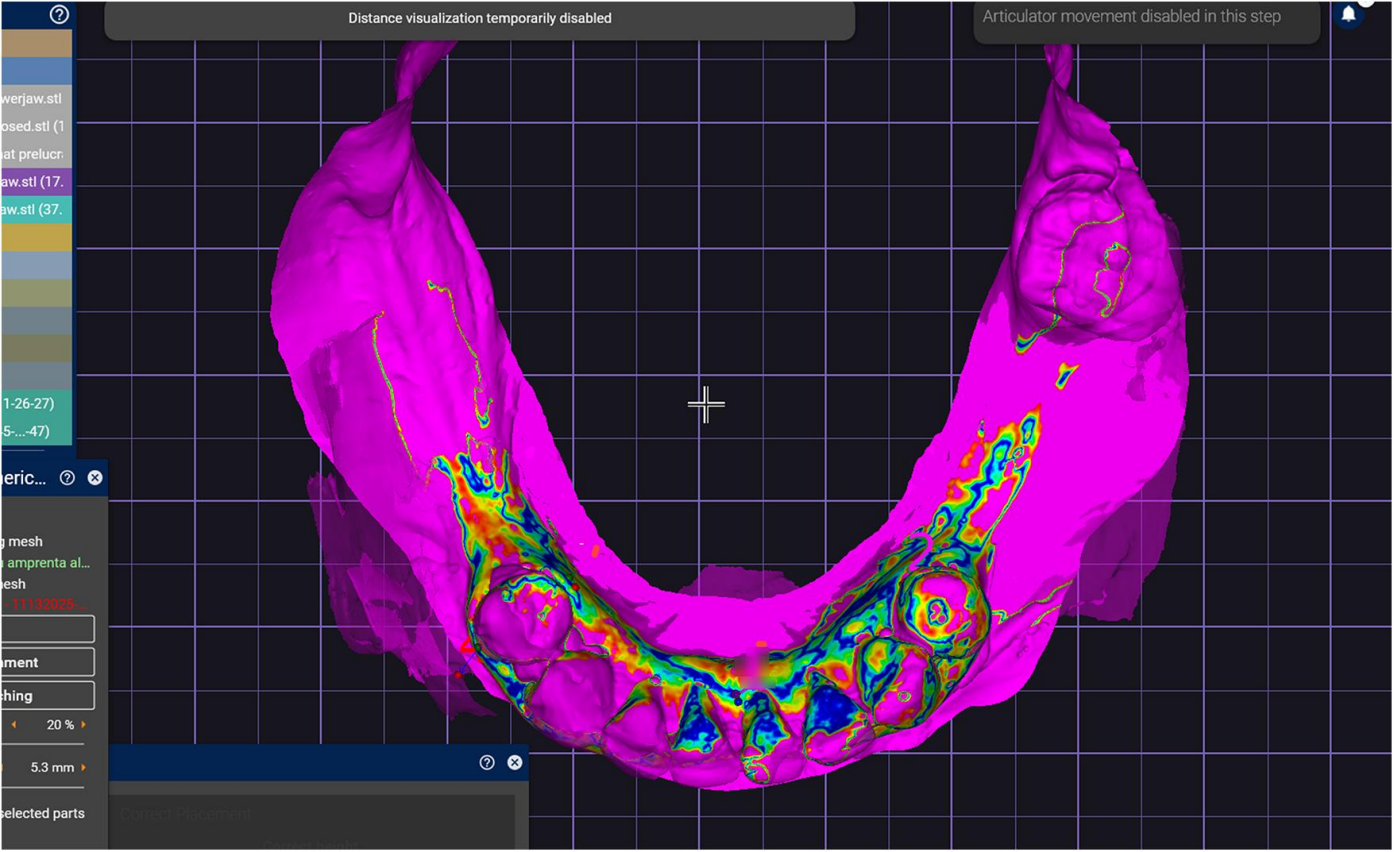
The intraoral scan and generated mesh superimposed in the design software based on reference areas represented by frontal lower teeth and attached gingiva.

**Figure 12:**
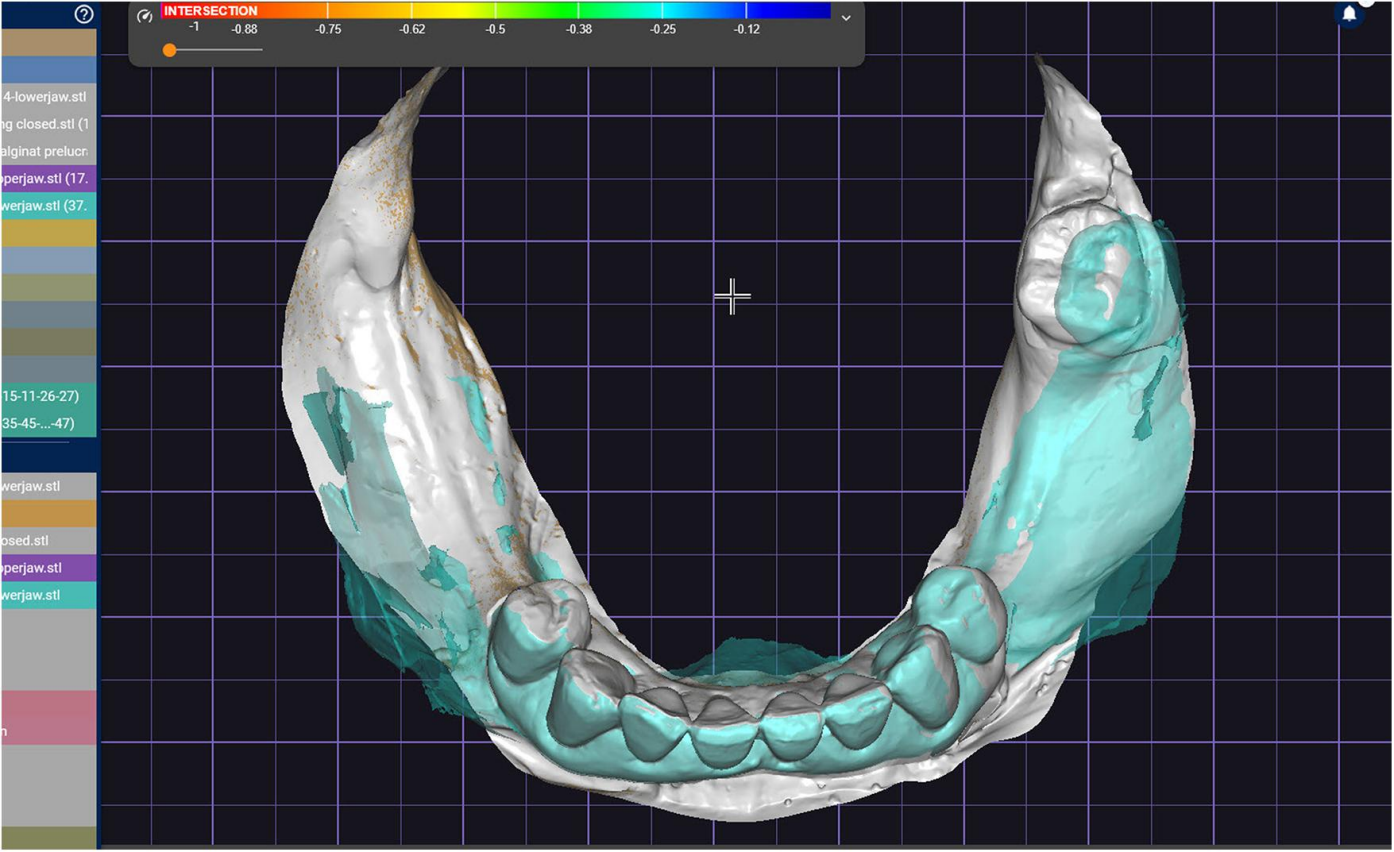
The superimposed meshes prepared for stitching selected areas.

**Figure 13:**
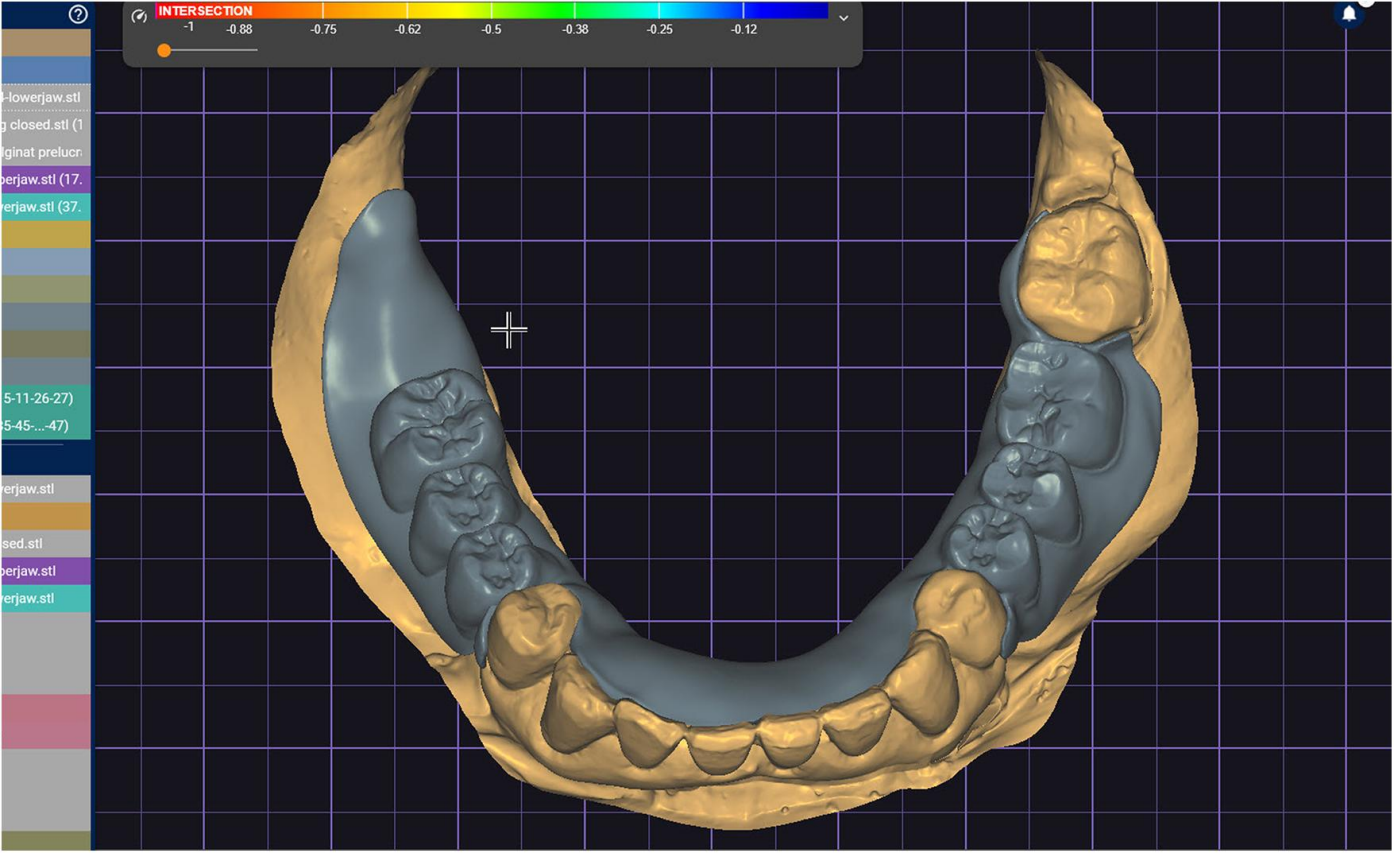
The final undistorted digital mesh of lower jaw obtained based on combined intraoral scan and digitized conventional impression and the designed validation try-in placed on digital mesh.

**Figure 14:**
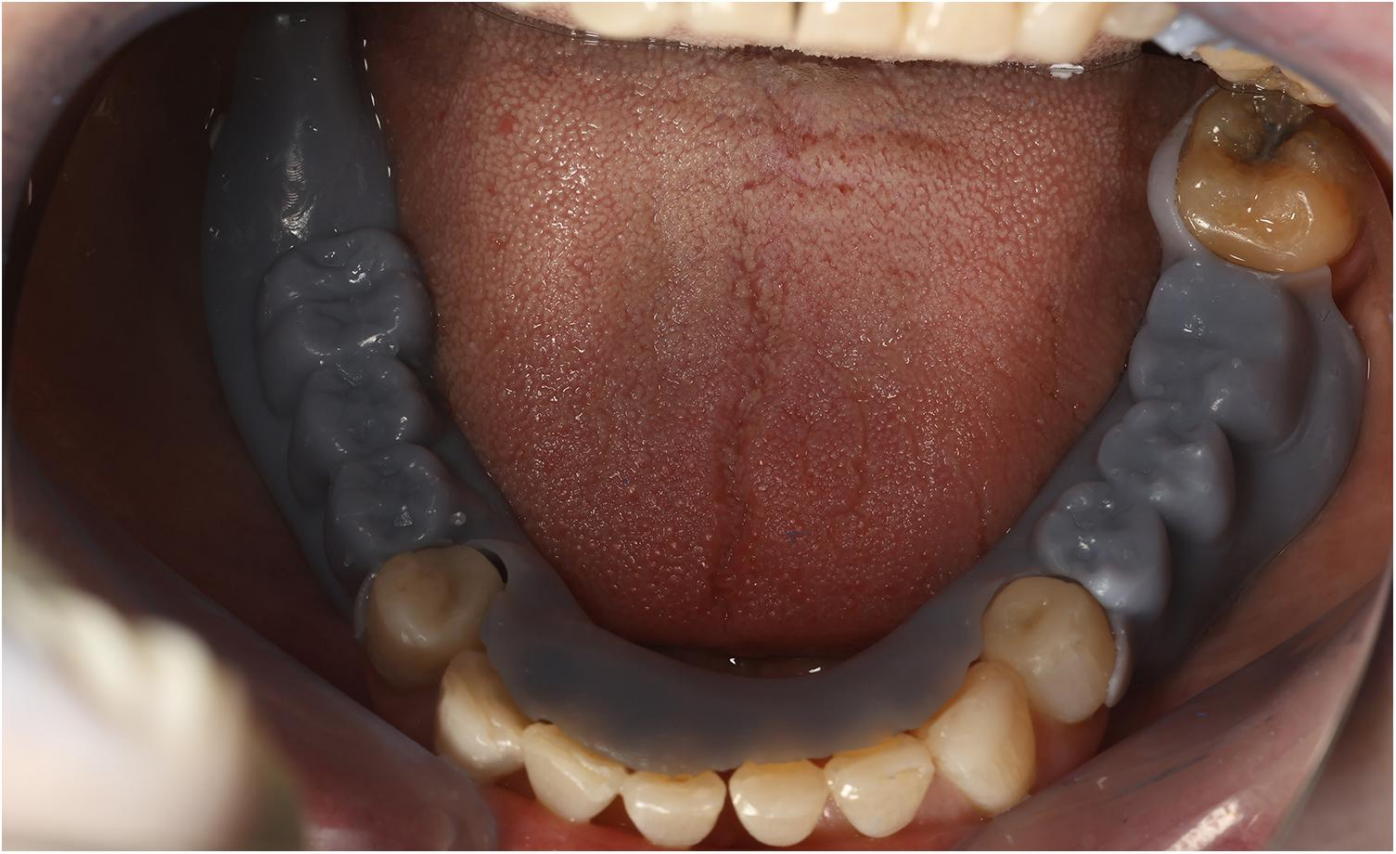
The 3D printed try-in mouth validation.

**Figure 15:**
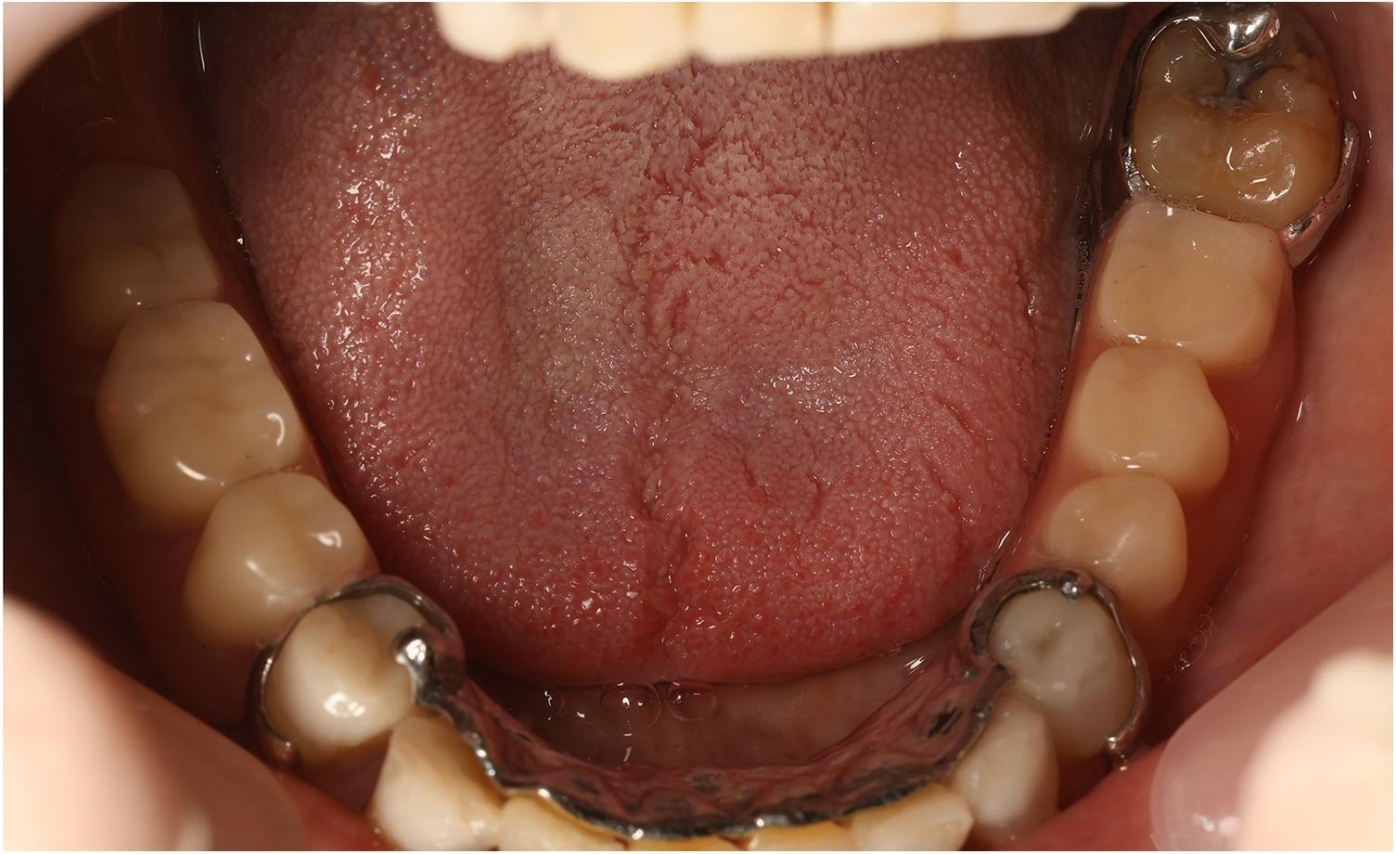
The final prosthesis placed on the lower jaw.

1. A challenging intraoral scanning scenario is presented due to the terminal mandibular edentulism on the right side and the extensive partial edentulism on the left. In quadrant 4, the proximity of the mucogingival junction to the edentulous ridge makes scanning of the ridge and the piriform tubercle particularly difficult. In quadrant 3, the wide area of mobile mucosa prevents the position of the second molar from being considered reliable (Figure 8).
2. An immediate alginate impression was taken using a stock universal tray. The borders of the mobile mucosa can also be transferred onto the impression using a mucosal marker. This marking can be visualized on a color scan and subsequently defined on the impression scan (Figure 9).
3. The relevant portion of the scan is digitally sectioned and the mesh normals are inverted. The impression exhibits localized positive artifacts caused by entrapped air bubbles (Figure 10).
4. The positive artifacts are digitally corrected, and the final borders are established on the mesh generated from the physical impression. It is evident that the intraoral scan of the impression exhibits deficiencies in the negative areas—corresponding to the teeth—where the intraoral scanner is unable to adequately capture details.
5. Within the design software, the two scans are superimposed using shared reference structures located on the remaining teeth and the attached gingiva (Figure 11).
6. The useful regions from the intraoral scan (the digital impressions of the positive elements—namely the teeth) are selected, along with the relevant areas from the impression scan (the digital impressions of the edentulous ridges and other regions susceptible to stitching-related distortion).
7. The final mesh obtained by merging the two partial scans is imported into the design software (exocad; exocad GmbH; Darmstadt, Germany). In the present case, there has been used the digital impression of the teeth from the intraoral scan, the edentulous ridges from the impression scan, and the morphology of the second molar, which was transferred from the intraoral scan and repositioned according to its recorded location on the impression scan (Figure 12).
8. The resulting digital mesh prepared to be validated intraorally in further steps.
9. In the design software (exocad; exocad GmbH; Darmstadt, Germany) a validation try-in has been designed (Figure 13).
10. The try-in has been printed with a 3D printer and intraorally validated (Figure 14).
11. The completed prosthesis has been evaluated on the model and delivered to the patient (Figure 15).

## 3 Results

Using the described protocol, the clinician obtains a STL file integrating dentate surfaces from the IOS with stable mucosal geometry from the digitized impression. The complete process is performed chairside without a desktop scanner, and the final model is ready for CAD design of RPD frameworks or other digital procedures.

In the example presented, the retromolar pad is only partially represented due to clinical limitations during intraoral scanning. This is a case-specific limitation rather than a restriction of the workflow itself. For routine RPD planning, clinicians are advised to extend both the IOS and the alginate impression coverage to include the complete retromolar pad region.

## 4 Discussion

This article presents a chairside hybrid workflow that merges an IOS optical scan with a digitized irreversible hydrocolloid impression—both captured with the same IOS device—to produce an working full-arch mandibular model in a single visit. In line with the rationale outlined in the Introduction, the approach seeks to retain high-fidelity dental surfaces from IOS while replacing potentially distorted edentulous mucosa with impression-derived geometry, thus addressing weaknesses of IOS over wide mucosal spans (8, 10–12).

Against this background, we position our approach relative to the existing evidence. Full-arch IOS performance is known to depend on scanner hardware/software, scanning pattern, arch, and the presence of auxiliary landmarks (10, 13–16). Clinical and *in-vitro* data show that modifying the scanning strategy can alter trueness and precision (13, 14), and that artificial landmarks/scanning aids can improve registration stability (15, 16). At the same time, feasibility studies of edentulous scanning highlight variable accuracy across devices and protocols (8–12). Our workflow is conceptually aligned with earlier hybrid techniques that combine optical and conventional impressions (e.g., windowed trays, functional impressions) for flabby tissue management (17, 18), but the main differences consist in generating a new STL file containing a final mesh from two previous impressions, being entirely chairside (no desktop scanning), in formalizing a sectioning curves strategy around residual dentition, and in explicitly defining a mesh inversion step for the impression surface. Standardized quality-control steps further distinguish this method from prior reports.

Translating these observations into practice, we highlight the operational implications of the proposed workflow. The workflow:

- Is performed in a single appointment;
- Uses a single IOS for both intraoral capture and impression digitization;
- Produces a standard STL compatible with common CAD platforms for RPD and guide design.

Through the present paper the clinical and numerical superiority of the hybrid model is not claimed. Future research could include quantitative trueness analyses using root-mean-square deviation maps, operator-repeatability assessments, and clinical fit evaluations of RPD frameworks fabricated from composite models.

Beyond feasibility, outcomes depend on a set of technical factors that warrant control. Key parameters likely to affect outcomes include:

- Impression quality (material selection, tray fit, and impression technique);
- Local best-fit constraints during targeted alignment to avoid propagating IOS errors into impression-derived regions;
- The placement of separation areas which governs where substitution occurs.

These considerations align with determinants of IOS accuracy reported for edentulous arches and with factors known to influence digital fit in RPD frameworks (10, 13–16).

Despite these measures, several limitations must be acknowledged. This work does not include a quantitative comparison with alternative methods. The hybrid model's trueness relative to IOS-only or laboratory-digitized casts was not measured; nor were precision/repeatability across operators, time metrics, or clinical fit outcomes. The approach still relies on a conventional impression, so it is not fully digital. Finally, generalizability to other IOS brands, scanning patterns, and maxillary arches remains to be established (13–16, 19).

Given these limitations, the next question is what this protocol means for clinical practice. In partially edentulous mandibles—where fewer rigid landmarks and mobile mucosa complicate full-arch IOS—the composite model can improve the predictability of path-of-insertion analyses, relief planning over mobile mucosa, and the placement of finishing lines for major connectors. Indirectly, higher model fidelity may support more consistent clasp engagement and connector adaptation, consistent with clinical and *in-vitro* evidence on digital RPD frameworks (21–24). For implant-assisted prosthetic planning, reducing soft-tissue distortion over long spans may improve guide design that depends on soft-tissue referencing (10–12).

To underpin these implications, targeted studies with defined metrics and endpoints are needed. Validation should examine the contribution of artificial landmarks/scanning aids (15, 16) and collect clinical endpoints such as framework seating, adjustment time, insertion time, and patient-reported comfort (22–24).

The protocol requires approximately 10–15 additional minutes beyond a standard IOS and 30 to 60 additional minutes to design and print a 3D try-in, as previously shown in described clinical report. Compatibility across different IOS brands and CAD platforms may vary and may require workflow adaptation. Sources of potential error could include inadequate sectioning lines and inadvertent global alignment across teeth and attached mucosa. Taken together, these elements delineate the strengths and novelty of the approach presented. The strengths of this work are the entirely chairside execution, use of a single IOS for both captures, explicit mesh inversion of the impression negative, and a reproducible segmentation procedure that confines substitution to pre-defined mucosal patches. Framed against the variability of edentulous IOS accuracy (10, 13–16) and the practical needs of digital RPD workflows (21–24), this represents a pragmatic, office-based pathway that is straightforward to adopt and to test prospectively.

## 5 Conclusion

A practical chairside workflow that combines an IOS optical impression with a digitized irreversible hydrocolloid impression can produce an undistorted, full-arch mandibular STL model in a single visit. The model is suitable for digital RPD framework design and other CAD/CAM tasks and may reduce the need for desktop scanning in routine practice.

## Data Availability

The original contributions presented in the study are included in the article/Supplementary Material, further inquiries can be directed to the corresponding author.
